# A Portable Diagnostic Assay, Genetic Diversity, and Isolation of Seoul Virus from *Rattus norvegicus* Collected in Gangwon Province, Republic of Korea

**DOI:** 10.3390/pathogens11091047

**Published:** 2022-09-14

**Authors:** Kyungmin Park, Seung-Ho Lee, Jongwoo Kim, Jingyeong Lee, Geum-Young Lee, Seungchan Cho, Juyoung Noh, Jeewan Choi, Juwon Park, Dong-Hyun Song, Se Hun Gu, Hyeongseok Yun, Jung-Eun Kim, Daesang Lee, Il-Ung Hwang, Won-Keun Kim, Jin-Won Song

**Affiliations:** 1 BK21 Graduate Program, Department of Biomedical Sciences, Korea University College of Medicine, Seoul 02841, Korea; 2 Department of Microbiology, College of Medicine, Korea University, Seoul 02841, Korea; 3 Chem-Bio Technology Center, Agency for Defense Development, Daejeon 34186, Korea; 4 Republic of Korea Armed Forces Medical Command, Seongnam 13415, Korea; 5 The Fifth Preventive Medicine Unit of Republic of Korea Army, Pocheon 11132, Korea; 6 Department of Orthopaedic Surgery, Sheikh Khalifa Specialty Hospital, Seoul National University Hospital, Seoul 02841, Korea; 7 Department of Microbiology, College of Medicine, Hallym University, Chuncheon 24252, Korea; 8 Institute of Medical Research, College of Medicine, Hallym University, Chuncheon 24252, Korea

**Keywords:** Seoul virus, hemorrhagic fever with renal syndrome, Biomeme system, portable diagnostic assay, molecular diagnosis, next-generation sequencing, whole-genome sequencing, genetic diversity, hantavirus isolation

## Abstract

Seoul virus (SEOV), an etiological agent for hemorrhagic fever with renal syndrome, poses a significant public health threat worldwide. This study evaluated the feasibility of a mobile Biomeme platform for facilitating rapid decision making of SEOV infection. A total of 27 *Rattus norvegicus* were collected from Seoul Metropolitan City and Gangwon Province in Republic of Korea (ROK), during 2016–2020. The serological and molecular prevalence of SEOV was 5/27 (18.5%) and 2/27 (7.4%), respectively. SEOV RNA was detected in multiple tissues of rodents using the Biomeme device, with differences in Ct values ranging from 0.6 to 2.1 cycles compared to a laboratory benchtop system. Using amplicon-based next-generation sequencing, whole-genome sequences of SEOV were acquired from lung tissues of Rn18-1 and Rn19-5 collected in Gangwon Province. Phylogenetic analysis showed a phylogeographical diversity of rat-borne orthohantavirus collected in Gangwon Province. We report a novel isolate of SEOV Rn19-5 from Gangwon Province. Our findings demonstrated that the Biomeme system can be applied for the molecular diagnosis of SEOV comparably to the laboratory-based platform. Whole-genome sequencing of SEOV revealed the phylogeographical diversity of orthohantavirus in the ROK. This study provides important insights into the field-deployable diagnostic assays and genetic diversity of orthohantaviruses for the rapid response to hantaviral outbreaks in the ROK.

## 1. Introduction

Hantaviruses (order *Bunyavirales*, family *Hantaviridae*, and genus *Orthohantavirus*) are enveloped, single-stranded, negative-sense RNA viruses, consisting of large (L), medium (M), and small (S) genome segments [1]. The tripartite RNA segments encode an RNA-dependent RNA polymerase, two surface glycoproteins (G_n_ and G_c_), and a nucleocapsid (N) protein, respectively [2]. The natural reservoirs of hantaviruses include rodents (Rodentia), bats (Chiroptera), and insectivores (Soricomorpha), supporting the coevolution between viruses and their hosts [3,4]. In humans, orthohantaviruses are zoonotic pathogens that account for hemorrhagic fever with renal syndrome (HFRS) in Eurasia and hantavirus cardiopulmonary syndrome in the Americas [5]. HFRS is mainly caused by Hantaan virus (HTNV), Seoul virus (SEOV), Dobrava–Belgrade virus, and Puumala virus (PUUV), which are transmitted to humans via inhalation of contaminated excreta, aerosols, or rarely, bites from infected rodents [6,7].

SEOV-related HFRS is responsible for approximately 20% of clinical cases with a mortality rate of <1% [8]. SEOV infection occurs in Asia, Europe, the Americas, and Africa owing to the global distribution of reservoir hosts (brown rat, *Rattus norvegicus*; black rat, *R. rattus*) [9]. Previous studies demonstrated SEOV outbreaks in patients directly exposed in the vicinity of infected wild rats in the United States of America (USA) [10,11,12]. SEOV-induced HFRS cases were reported in humans engaged in occupation handling infected laboratory rats or tissue culture lines in Japan and the United Kingdom (UK) [13,14]. Recent clinical cases of autochthonous SEOV infection have been primarily associated with contact with pet rats and feeder rats in the USA, UK, France, and the Netherlands [15,16,17,18]. SEOV-induced HFRS cases are considered an underestimation worldwide due to the limited knowledge in the transmission and epidemiology of viruses and their hosts [19].

The Franklin system (Biomeme, Philadelphia, PA, USA) is a handheld portable real-time polymerase chain reaction (PCR) instrument that weighs approximately 1.4 kg with a rechargeable battery [20,21]. The quantitative PCR (qPCR) assay with Biomeme technologies offers a rapid and sensitive molecular diagnosis of infectious agents, including African swine fever virus, foot-and-mouth disease virus, Ebola virus (EBOV), and severe acute respiratory syndrome coronavirus 2 (SARS-CoV-2) in animal and human samples [22,23,24,25]. Here, we evaluated whether the handheld Biomeme platform could be used for a molecular diagnostic assay of SEOV to facilitate rapid decision making in point-of-care testing (POCT).

The acquisition of viral genomic sequences plays an important role in the diagnosis, characterization, epidemiological surveillance, and risk mitigation of hantaviral outbreaks by tracking the precise causative agents and putative infection sites. The high molecular identities of genome sequences of PUUV derived from humans and voles revealed clear phylogeographical association and spatial evolution of each virus clade in Germany [26]. Comparative genomic analyses of HTNV elicited epidemiological links of patients with HFRS and natural reservoir *Apodemus agrarius* captured at the putative exposed sites in the Republic of Korea (ROK) [27]. The serological and molecular investigation of small mammals demonstrated the epidemiological characteristics and risk of SEOV carried by urban rodents in Seoul Metropolitan City, ROK [28]. Multiplex-PCR-based next-generation sequencing (NGS) elucidated the global phylogenetic shape of SEOV from clinical and reservoir host specimens [29]. However, our knowledge of the epidemiologic dynamics of SEOV infection is limited owing to the lack of viral sequences.

In this study, a small-scale epidemiological survey of wild rodents was conducted in the rat population from 2016 to 2020 to determine the serological and molecular prevalence of SEOV in the ROK. We report the development of a TaqMan probe-based one-step qPCR assay with a portable Biomeme system for real-time identification of SEOV. Whole-genome sequencing and isolation of SEOV, Gangwon Province, delineate the phylogeographic diversity in the ROK. These findings provide important insights into the field-deployable diagnostic assays and the genetic diversity of rat-borne orthohantavirus for the rapid response to hantaviral outbreaks in the ROK. 

## 2. Materials and Methods

### 2.1. Ethics Statement

Animal trapping was approved in accordance with the ethical guidelines of the Korea University Institutional Animal Care and Use Committee (KUIACUC #2016-49 and 2020-14). Autopsy was performed at an animal biosafety level 3 facility at Korea University.

### 2.2. Sample Collection

Small mammals were collected using Sherman live traps (8 cm × 9 cm × 23 cm; H. B. Sherman, Tallahassee, FL, USA) from multiple sites including Seoul Metropolitan City and Gangwon Province (Cheorwon-gun and Chuncheon-si), ROK, during 2016–2020. The trapping and transportation procedures were described in a previous study [30]. The positive traps were sequentially numbered, identified by morphological characteristics, placed in a sealed container, and then transported to Korea University, Seoul, ROK. Additionally, two *R. norvegicus*, two *A. agrarius*, two *Crocidura lasiura*, and two *Mus musculus*, captured from Gangneung-si in Gangwon Province, were provided by the HFRS vector surveillance program of the ROK Army in 2019. The serum samples and tissues (lung, liver, kidney, and spleen) of captured animals were collected aseptically and stored at −80 °C until use.

### 2.3. Mitochondrial DNA Analysis

Total DNA was extracted from the liver tissues of rodents using a High Pure PCR template preparation kit (Roche, Basel, Switzerland). The mitochondrial DNA cytochrome *b* gene was amplified using the conventional PCR method [31]. The obtained sequences were deposited in GenBank (accession numbers: OK746255-OK746256).

### 2.4. Indirect Immunofluorescence Assay (IFA)

The heart fluids of rats were initially diluted 1:2 with phosphate-buffered saline (PBS), placed in SEOV-infected Vero E6 cells fixed with cold acetone for 10 min, and incubated at 37 °C for 30 min. The plates were washed with PBS and distilled water. The slides were treated with fluorescein isothiocyanate-conjugated goat antibody to rat immunoglobulin G (IgG; ICN Pharmaceuticals, Laval, Quebec, Canada). The cells were incubated at 37 °C for 30 min and washed with PBS and distilled water. The virus-specific fluorescence was examined using a fluorescence microscope (Carl Zeiss, Berlin, Germany).

### 2.5. Reverse Transcription-PCR (RT-PCR)

Total RNA was extracted from the lung tissues of rats using TRI Reagent Solution (Ambion, Austin, TX, USA) according to the manufacturer’s instructions. cDNA was synthesized using a High Capacity RNA-to-cDNA kit (Applied Biosystems, Foster City, CA, USA) with OSM55 (5′-TAG TAG TAG ACT CC-3′). PCR conditions and oligonucleotide primers were previously described [32].

### 2.6. qPCR

qPCR was performed using the TOPreal™ One-step RT qPCR Kit (Enzynomics, Daejeon, ROK) according to the manufacturer’s instructions. The optimal reaction mixture contained 1 µL of the RNA template, 1 µL of TaqMan probe (10 nM), 1 µL of forward and reverse primers (each 10 nM), 5 µL of TOPreal One-step RT-qPCR kit mix, and 12 µL of nuclease-free water in a final volume of 20 µL. The primer and probe sequences were SEOV-SF-1 (forward primer), 5′-GAC AGG ATT GCA GCA GGG AA-3′, SEOV-SR-1 (reverse primer): 5′-CGG CTC TAC CCC TGT AGG ATC-3′, and SEOV-SP-1 (probe): 5′-FAM-AAC ATC GGG CAA GAC CG-MGB-3′. The PCR was performed in triplicate on a Biomeme Franklin Three Real-Time PCR thermocycler system (Biomeme) and a Quantstudio 5 Flex Real-Time PCR System (Applied Biosystems) using the following cycling conditions: 30 min at 50 °C and 10 min at 95 °C, followed by 45 cycles of 5 s at 95 °C and 30 s at 60 °C. The cutoff value is 40.

### 2.7. Multiplex-PCR-Based NGS

cDNA was amplified using SEOV-specific primer mixtures and Solg 2X Uh-Taq PCR Smart Mix (Solgent, Daejeon, ROK) according to the manufacturer’s instructions. The PCR program and primer information for DNA libraries were described in the previous study [29]. The amplified products were prepared using a TruSeq Nano DNA LT sample preparation kit (Illumina, San Diego, CA, USA) according to the standard protocol. DNA libraries were sheared using an M220 focused ultrasonicator (Covaris, Woburn, MA, USA). The libraries were A-tailed, ligated to indexes and adaptors, and enriched by PCR. The quality and concentration of the libraries were assessed using the Agilent DNA 1000 chip on a bioanalyzer (Agilent Technologies, Santa Clara, CA, USA). The samples were sequenced on a MiSeq benchtop sequencer (Illumina) with 2 × 150 bp using a MiSeq reagent kit v2 (Illumina). The raw data were trimmed from the adaptor sequences using CLC Genomics Workbench version 7.5.2 (Qiagen, Hilden, Germany). The filtered reads were mapped onto the reference sequences of SEOV 80-39, and consensus sequences were extracted. 

### 2.8. Rapid Amplification of cDNA Ends (RACE) PCR

To complete the 3′ and 5′ terminal genome sequences of SEOV, RACE PCR was performed using a RACE System for Rapid Amplification of cDNA Ends, Version 2.0, (Invitrogen, Carlsbad, CA, USA), according to the manufacturer’s specifications. The obtained genome sequences of SEOV were deposited in GenBank (accession numbers: OK746249-OK746254).

### 2.9. Phylogenetic Analysis

The genome sequences of SEOV were aligned using the Clustal W tool in the Lasergene program, version 5 (DNASTAR, Madison, WI, USA). Phylogenetic trees were generated using the Markov chain Monte Carlo (MCMC) method, MrBayes 3.2.7a, with optimal evolutionary models estimated using the maximum-likelihood statistical method in MEGA 7 [33,34]. Bayesian analysis consisted of independent duplicate runs with 10 million MCMC generations sampled every 100 generations with a 25% burn-in. The phylogeny was visualized using FigTree 1.4.4 (available at: http://tree.bio.ed.ac.uk/software/figtree; accessed on 14 September 2022).

### 2.10. Cell Lines

Vero E6 cells (ATCC, #DR-L2785) were purchased from ATCC. The cells were maintained in Dulbecco’s modified Eagle’s medium (DMEM) supplemented with 10% fetal bovine serum (FBS), 1% HEPES buffer (Lonza, Basel, Switzerland), 1% L-glutamine (Lonza), and 0.1% gentamicin (Gibco, Life Technologies, Carlsbad, CA, USA). The cultures were incubated at 37 °C in an incubator with 5% CO_2_.

### 2.11. Virus Isolation

SEOV-positive lung tissues were ground in DMEM. After centrifugation, the supernatant was inoculated into the Vero E6 cells. After 1.5 h of adsorption, the excess inoculum was discarded, and the viral suspension was replaced with 5.5 mL of DMEM containing 5% FBS, 1% HEPES buffer (Lonza), 1% L-glutamine (Lonza), and 0.1% gentamicin (Gibco). The cells were incubated at 37 °C in an incubator with 5% CO_2_ and passaged at 2 weeks intervals. At each passage level, the cell culture was tested for the presence of SEOV antigen and RNA using the IFA and RT-PCR, respectively. 

### 2.12. Plaque Assay

Vero E6 cells were seeded in 6-well plates at a density of 2 × 10^6^ cells per well. After overnight incubation at 37 °C with 5% CO_2_, the cells were washed twice with PBS and inoculated with 10-fold serially diluted SEOV. After a 1.5 h absorption at 37 °C with constant shaking, the monolayer was overlaid with the overlay medium and medium-melting-point agarose mix (2:1 ratio). The plaques were incubated at 37 °C for 14 days and then visualized by staining the cells with 5% neutral red solution (Sigma-Aldrich, Burlington, VT, USA). 

### 2.13. Statistical Analysis

Student’s *t*-test was conducted to determine significant differences in performance between Franklin (Biomeme) and QuantStudio 5 (Applied Biosystems) platforms.

## 3. Results

### 3.1. Serological and Molecular Prevalence of SEOV

**Figure 1:**
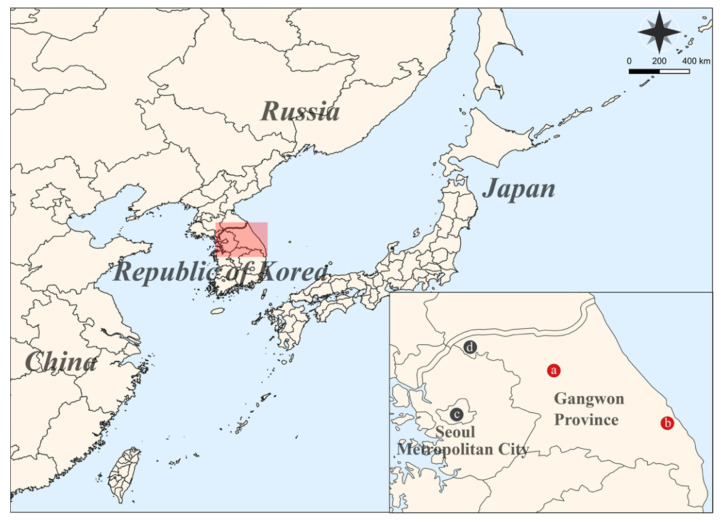
A geographic map of trapping sites for small mammals collected in Seoul Metropolitan City and Gangwon Province, Republic of Korea (ROK), from 2016 to 2020. The red circles indicate the SEOV RNA-positive areas: a, Chuncheon-si; b, Gangneung-si in Gangwon Province, respectively. The black circle represents the locations where no SEOV RNA was confirmed: c, Seoul Metropolitan City; d, Cheorwon-gun in Gangwon Province. The map was created using a Quantum Geographical Information System (QGIS) 3.10 for Mac and modified in Adobe Illustrator CC 2019.

A total of 27 *R. norvegicus* were collected from multiple locations, including Seoul Metropolitan City, Cheorwon-gun, Chuncheon-si, and Gangneung-si in the ROK, 2016–2020 (Figure 1). Geographic locations of the trapping are shown in Table S1. The serological prevalence of anti-SEOV IgG was 5/27 (18.5%), of which 3/23 (13.0%), 1/1 (100%), and 1/2 (50%) were detected in Seoul Metropolitan City, Chuncheon-si, and Gangneung-si, respectively; no seropositive rats were observed in Cheorwon-si (Table 1 and Figure S1). The molecular prevalence of SEOV RNA was 2/27 (7.4%), including 1/1 (100%) in Chuncheon-si and 1/2 (50%) in Gangneung-si. The positivity of SEOV RNA was undetectable in the rodents captured in Seoul Metropolitan City and Cheorwon-gun.

### 3.2. Comparison between Handheld and Benchtop qPCR for SEOV

**Figure 2:**
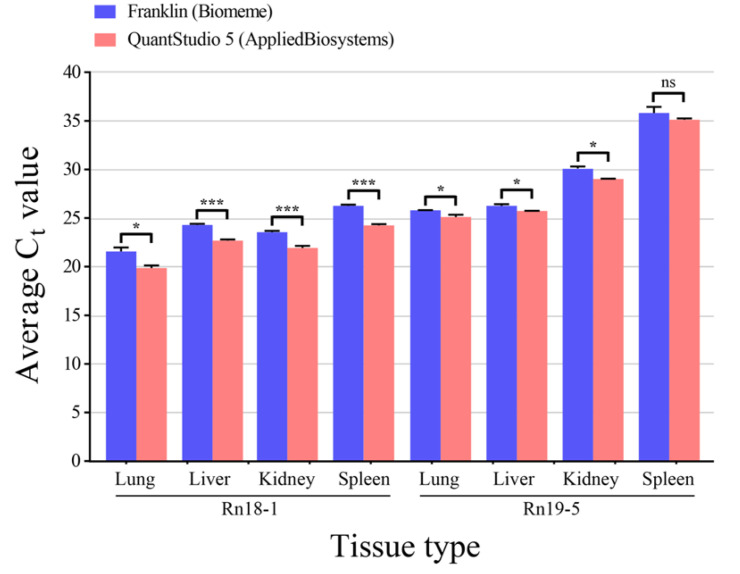
Comparison of Cycle threshold (Ct) values between two qPCR platforms for Seoul virus (SEOV) RNA loads in various rodent tissues. qPCR was performed in triplicate on both Biomeme Franklin and Applied Biosystems QuantStudio 5. Ct values were determined for the S segment of SEOV RNA in the lung, liver, kidney, and spleen tissues acquired from the infected rats. SEOV RNA was not detectable in negative control group. The cutoff value is 40. Asterisk (*) indicates the significant difference of Ct values between qPCR machines, using the student’s *t*-test (* *p* < 0.05; *** *p* < 0.001; ns: non-significant). Ct, cycle threshold; S, small.

To compare the relative sensitivity between handheld and benchtop qPCR systems for SEOV analysis, qPCR was performed on the lung, liver, kidney, and spleen tissues (Figure 2). SEOV RNA was detected in all tissues of rodents, Rn18-1 and Rn19-5, using two qPCR machines. RT-PCR negative samples showed that SEOV RNA was not detectable. In all tissue types, the cycle threshold (Ct) values generated from the Biomeme portable device were consistently higher than those of the laboratory benchtop system. The differences in Ct values ranged from 0.6 to 2.1 cycles between the Biomeme device and QuantStudio 5 system with the significance (*p* < 0.05), except for the Rn19-5 spleen tissue.

### 3.3. Whole-Genome Sequencing of SEOV

Using multiplex PCR-based NGS, nearly whole-genome sequences of SEOV were obtained from the lung tissues of Rn18-1 and Rn19-5 captured in Gangwon Province, ROK. The coverage of the genomic sequences of SEOV was 97.3–99.6% for L segments, 96.5–98.9% for M segments, and 90.0–97.3% for S segments, respectively (Table 2). The mean numbers of mapped viral reads and depth are shown in Table S2. The 3′ and 5′ termini sequences were obtained by RACE PCR. The 3´ and 5´ end sequences of three SEOV segments were 5′-GGA GUC UAC UAC UA-3′ and 5′-UAG UAG UAU GCU CC-3′. The nucleotide identity of SEOV Rn18-1 and Rn19-5 tripartite genomes was 97.7–98.4% for the L segment, 97.3–98.6% for the M segment, and 98.2–98.6% for the S segment with prototype SEOV 80–39. The similarity in the amino acid levels of SEOV was 99.5% for the L segment, 98.9–99.2% for the M segment, and 99.8–100% for the S segment, respectively.

### 3.4. Phylogenetic Analysis of SEOV

**Figure 3:**
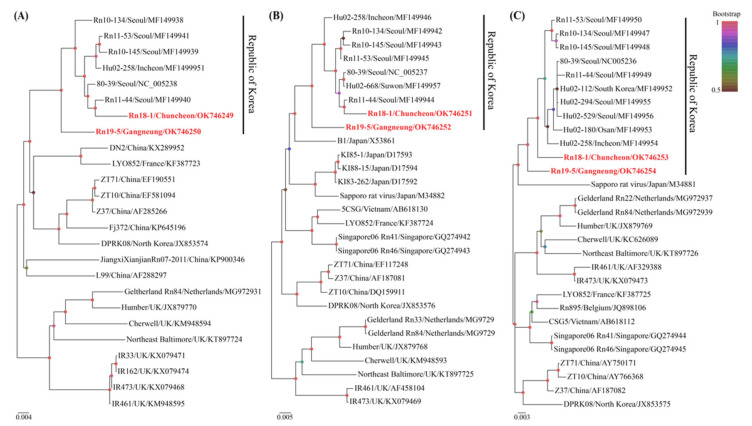
Phylogenetic analysis of the L, M, and S segments of Seoul virus (SEOV) from wild rats collected in Gangwon Province, Republic of Korea. The phylogenies were generated using Markov chain Monte Carlo (MCMC) methods MrBayes 3.2.7a with best-fit evolutionary models GTR+G (for L segment), GTR+G+I (for M segment), and HKY+G (for S segment) based on the SEOV (**A**) L (1–6,530 nt), (**B**) M (1–3,651 nt), and (**C**) S (1–1,769 nt) segments, respectively. The red letter indicates genomic sequences of SEOV obtained in this study. The bootstrap score (0.5–1) was illustrated at the tip of each node. L, large; M, medium; S, small.

Phylogenetic analysis showed that SEOV harbored by *R. norvegicus* in Gangwon Province were closely related to those of rat- and human-derived viral genomes collected in ROK (Figure 3). In particular, the SEOV Rn19-5 strain from Gangneung-si formed a distinct genetic lineage with all other viral genomes, ROK. The phylogenies of the L and M segments of SEOV Rn18-1 from Chuncheon-si shared a common ancestor with the SEOV Rn11-44 collected in Seoul Metropolitan City, whereas the S segment formed an independent genetic group in SEOV, ROK.

### 3.5. Virus Isolation

SEOV was isolated from the lung tissues of Rn19-5 using a cell-culture-based method. The first isolate of SEOV was confirmed at 42 days post-inoculation, and the number of infectious particles was 3.2 × 10^2^ PFU/mL (Figure S2).

## 4. Discussion

The establishment of a rapid and sensitive diagnostic assay is required for the detection and characterization of SEOV [6,9]. Numerous serological and molecular methods have been developed for the diagnosis, including enzyme-linked immunosorbent assay, IFA, loop-mediated isothermal amplification, and RT-PCR [35,36,37,38]. qPCR has several advantages of being faster, sensitive, and reproducible, with a precise quantitation of viral loads [39,40,41,42]. However, the application of qPCR assays for pathogen detection has been limited by thermocycler size, testing consumables, and maintaining power in outdoor environments. Physicians and epidemiologists need a portable diagnostic approach for rapid decision making in the POCT of patients. The Biomeme Franklin system, a mobile qPCR thermocycler, enables the monitoring of multiple fluorophores with real-time diagnosis, making it an appropriate device to be used in field diagnostics for the detection of infectious diseases [20,43]. The portable point-of-encounter assay showed the early detection of EBOV with comparable specificity and sensitivity to laboratory-based systems [24]. Onyilagha et al. demonstrated the application of a field-deployable point-of-care real-time qPCR with a mobile Biomeme system for rapid, specific, and sensitive detection of SARS-CoV-2 in animal and human samples [25]. In this study, we evaluated a TaqMan probe-based one-step qPCR assay using the Biomeme platform for real-time detection and quantification of SEOV. These results showed that the portable Biomeme device can be used to detect and monitor SEOV, with comparable performance to the laboratory-based qPCR system under laboratory testing conditions. However, further studies need to validate the performance of the assay from patients with SEOV-related HFRS in the field.

The generation of viral genomic sequences plays an important role in clinical diagnosis, patient precision management, epidemiological surveillance, and risk mitigation of virus outbreaks [44,45]. NGS is a robust tool for monitoring and tracking emerging viral infections to link viral genomic sequences and epidemiologic information. Genomic surveillance of NGS has been applied to understand the characteristics and transmission dynamics of zoonotic viruses including EBOV, Zika virus, Dabie bandavirus, and SARS-CoV-2 [46,47,48,49]. In a previous study, whole-genome sequences of HTNV generated from multiplex-PCR-based NGS enabled defining the phylogenetic association between patients with HFRS and reservoir hosts captured at potentially exposed sites in the ROK [50]. Recently, NGS-based active surveillance, including targeted animal trapping in the suspected locations of hantaviral outbreaks, demonstrated the phylogeographical correlation between patients with HFRS and HTNV to identify precise etiological agents and putative infection areas within a relatively short distance (5 km) [51]. The establishment of viral sequence-based surveillance revealed the putative epidemiological relationship of patients with infectious sources and the development of preventive strategies for mitigating HFRS incidences [27,52]. Here, we reported the full-length genome sequences of SEOV newly obtained from wild rats (*R. norvegicus*) collected in Gangwon Provinces (Chuncheon-si and Gangneung-si), enhancing the resolution of a phylogeographic map of orthohantaviruses for the sophisticated prevention and diagnosis of hantaviral outbreaks. To our knowledge, these findings are the first molecular evidence of rodent-borne SEOV circulating in the Gangwon Province, ROK. Tripartite genomes of SEOV Rn19-5 from Gangneung-si formed an independent phylogenetic lineage with other SEOV derived from the ROK, indicating the well-supported genetic diversity of rodent-borne hantaviruses in the restricted areas. Given that the brown rat (*R. norvegicus*) is ubiquitous, our findings suggest that continued and large-scale surveillance are needed to understand the evolutionary diversification and geographic distribution of SEOV in the ROK.

The isolation of viruses imparts well-characterized and cell-adapted strains that can be applied for the development of potential antiviral and vaccine candidates [53,54]. In 1980, the first SEOV isolation occurred from *R. norvegicus* captured in Seoul Metropolitan City, ROK [36]. Further SEOV isolation was conducted in China (1982), Japan (1983), Egypt (1983), the UK (1984), the USA (1984), and Germany (1988) [14,55,56,57,58,59]. Although hantaviruses are difficult to isolate using the cell-culture-based method, SEOV is the only hantavirus species isolated from four continents [9]. In this study, we report an isolation of SEOV Rn19-5 strain from wild rat captured in Gangwon Province, which is the second SEOV isolate in the ROK. Our findings will allow the evaluation of the infectivity and pathogenicity of the divergent SEOV strain, facilitating the development of antiviral and vaccine candidates in humans.

In conclusion, a real-time one-step qPCR assay was implemented to the mobile Biomeme system for the rapid diagnosis and quantification of SEOV. The complete-length genomic sequences of two SEOVs were newly recovered from wild *R. norvegicus* captured in Gangwon Province, ROK. The molecular evidence of SEOV improved the resolution of the phylogeographic map of orthohantaviruses for elaborated prevention and tracking of hantavirus outbreaks in the ROK. Finally, we report the isolate of SEOV Rn 19-5 from the brown rat collected in Gangwon Province. This study provides important insights into the field-deployable diagnostic assays and the phylogeographic diversity of rat-borne orthohantaviruses in response to hantaviral outbreaks in the ROK.

## Figures and Tables

**Table 1:** Serological and molecular prevalence of SEOV in *Rattus norvegicus* collected in the Republic of Korea, 2016–2020.

Characteristic	Number of Captured *R. norvegicus*	Seropositivity for Anti-SEOV IgG (%)	SEOV RNA Positivity (%)
Region (*n* = 27)			
Seoul Metropolitan City	23	3/23 (13.0)	0/23
Cheorwon-gun	1	0/1	0/1
Chuncheon-si	1	1/1 (100)	1/1 (100)
Gangneung-si	2	1/2 (50)	1/2 (50)
Sex (*n* = 27)			
Male	19	4/19 (21.1)	2/19 (10.5)
Female	8	1/8 (12.5)	0/8
Weight (*n* = 27)			
<50	10	1/10 (10)	0/10
51–100	11	2/11 (18.2)	0/11
101–150	3	0/3	0/3
151–200	3	2/3 (66.7)	2/3 (66.7)
Total	27	5/27 (18.5)	2/27 (7.4)

SEOV, Seoul virus; IgG, immunoglobulin G.

**Table 2:** Next-generation sequencing coverage of Seoul virus (SEOV) from rats captured in Gangwon Province, Republic of Korea, during 2016–2020.

Sample	Region	Origin	Anti-SEOV IgG Titer	Ct Value ^a^	SEOV Genomes, % Coverage ^b^		
					L Segment	M Segment	S Segment
Rn18-1	Chuncheon-si	Lung	1:256	21.6	97.3	96.5	90.0
Rn19-5	Gangneung-si	Lung	1:256	25.8	99.5	98.9	97.3

IgG, immunoglobulin G; Ct, cycle threshold; Rn, *Rattus norvegicus*; L, large; M, medium; S, small. ^a^; determined using Biomeme system-based quantitative PCR for the S segment of SEOV. ^b^; genome coverage was calculated using consensus sequence matching to the genome positions of the SEOV 80-39 strain.

## Data Availability

Not applicable.

## Supplementary Materials

The following supporting information can be downloaded at: https://www.mdpi.com/article/10.3390/pathogens11091047/s1, Figure S1: Detection of immunoglobulin G (IgG) antibodies against Seoul virus (SEOV) from heart fluids of *Rattus norvegicus* by indirect immunofluorescence assay (IFA); Figure S2: The plaque assay of SEOV Rn19-5 strain; Table S1: Trapping sites and GPS coordinates in this study; Table S2: Summary of mapped reads and depth of coverage for Seoul virus using next-generation sequencing in this study.

## References

[1] Vaheri A., Henttonen H., Voutilainen L., Mustonen J., Sironen T., Vapalahti O. (2013). Hantavirus infections in Europe and their impact on public health. Rev. Med. Virol..

[2] Laenen L., Vergote V., Calisher C.H., Klempa B., Klingström J., Kuhn J.H., Maes P. (2019). *Hantaviridae*: Current classification and future perspectives. Viruses.

[3] Guo W.P., Lin X.D., Wang W., Tian J.H., Cong M.L., Zhang H.L., Wang M.R., Zhou R.H., Wang J.B., Li M.H. (2013). Phylogeny and origins of hantaviruses harbored by bats, insectivores, and rodents. PLoS Pathog..

[4] Park K., Kim W.-K., Lee S.-H., Kim J., Lee J., Cho S., Lee G.-Y., No J.S., Lee K.H., Song J.-W. (2021). A novel genotype of *Hantaan orthohantavirus* harbored by *Apodemus agrarius chejuensis* as a potential etiologic agent of hemorrhagic fever with renal syndrome in Republic of Korea. PLoS Negl. Trop. Dis..

[5] Kruger D.H., Figueiredo L.T.M., Song J.-W., Klempa B. (2015). Hantaviruses—Globally emerging pathogens. J. Clin. Virol..

[6] Kabwe E., Davidyuk Y., Shamsutdinov A., Garanina E., Martynova E., Kitaeva K., Malisheni M., Isaeva G., Savitskaya T., Urbanowicz R.A. (2020). Orthohantaviruses, Emerging Zoonotic Pathogens. Pathogens.

[7] Hart C.A., Bennett M. (1999). Hantavirus infections: Epidemiology and pathogenesis. Microbes Infect..

[8] Kim Y.S., Ahn C., Han J.S., Kim S., Lee J.S., Lee P.W. (1995). Hemorrhagic fever with renal syndrome caused by the Seoul virus. Nephron.

[9] Clement J., LeDuc J.W., Lloyd G., Reynes J.M., McElhinney L., Van Ranst M., Lee H.W. (2019). Wild Rats, Laboratory Rats, Pet Rats: Global Seoul Hantavirus Disease Revisited. Viruses.

[10] Roig I.L., Musher D.M., Tweardy D.J. (2012). Severe pulmonary involvement in a case attributed to domestically acquired Seoul hantavirus in the United States. Clin. Infect. Dis.

[11] Woods C., Palekar R., Kim P., Blythe D., de Senarclens O., Feldman K., Farnon E.C., Rollin P.E., Albarino C.G., Nichol S.T. (2009). Domestically acquired seoul virus causing hemorrhagic fever with renal syndrome-Maryland, 2008. Clin. Infect. Dis.

[12] Shastri B., Kofman A., Hennenfent A., Klena J.D., Nicol S., Graziano J.C., Morales-Betoulle M., Cannon D., Maradiaga A., Tran A. (2019). Domestically Acquired Seoul Virus Causing Hemophagocytic Lymphohistiocytosis-Washington, DC, 2018. Open Forum Infect. Dis..

[13] Kawamata J., Yamanouchi T., Dohmae K., Miyamoto H., Takahaski M., Yamanishi K., Kurata T., Lee H.W. (1987). Control of laboratory acquired hemorrhagic fever with renal syndrome (HFRS) in Japan. Lab. Anim. Sci..

[14] Lloyd G., Jones N. (1986). Infection of laboratory workers with hantavirus acquired from immunocytomas propagated in laboratory rats. J. Infect..

[15] Swanink C., Reimerink J., Gisolf J., de Vries A., Claassen M., Martens L., Waegemaekers T., Rozendaal H., Valkenburgh S., Hoornweg T. (2018). Autochthonous Human Case of Seoul Virus Infection, the Netherlands. Emerg. Infect. Dis..

[16] Kerins J.L., Koske S.E., Kazmierczak J., Austin C., Gowdy K., Dibernardo A., Group C.S.V.I., Group C.S.V.I., Group S.V.W. (2018). Outbreak of Seoul virus among rats and rat owners—United States and Canada, 2017. Morb. Mortal. Wkly. Rep..

[17] Jameson L.J., Logue C.H., Atkinson B., Baker N., Galbraith S.E., Carroll M.W., Brooks T., Hewson R. (2013). The continued emergence of hantaviruses: Isolation of a Seoul virus implicated in human disease, United Kingdom, October 2012. Euro Surveill..

[18] Mace G., Feyeux C., Mollard N., Chantegret C., Audia S., Rebibou J.M., Spagnolo G., Bour J.B., Denoyel G.A., Sagot P. (2013). Severe Seoul hantavirus infection in a pregnant woman, France, October 2012. Euro Surveill..

[19] Li Y., Cazelles B., Yang G., Laine M., Huang Z.X.Y., Cai J., Tan H., Stenseth N.C., Tian H. (2019). Intrinsic and extrinsic drivers of transmission dynamics of hemorrhagic fever with renal syndrome caused by Seoul hantavirus. PLoS Negl. Trop. Dis..

[20] Marx V. (2015). PCR heads into the field. Nat. Methods.

[21] Thomas A.C., Tank S., Nguyen P.L., Ponce J., Sinnesael M., Goldberg C.S. (2020). A system for rapid eDNA detection of aquatic invasive species. Environ. DNA.

[22] Daigle J., Onyilagha C., Truong T., Le V.P., Nga B.T.T., Nguyen T.L., Clavijo A., Ambagala A. (2021). Rapid and highly sensitive portable detection of African swine fever virus. Transbound Emerg. Dis..

[23] Hole K., Nfon C. (2019). Foot-and-mouth disease virus detection on a handheld real-time polymerase chain reaction platform. Transbound Emerg. Dis..

[24] Figueroa D.M., Kuisma E., Matson M.J., Ondzie A.U., Bushmaker T., Seifert S.N., Ntoumi F., Escudero-Pérez B., Muñoz-Fontela C., Walzer C. (2021). Development and validation of portable, field-deployable Ebola virus point-of-encounter diagnostic assay for wildlife surveillance. One Health Outlook.

[25] Onyilagha C., Mistry H., Marszal P., Pinette M., Kobasa D., Tailor N., Berhane Y., Nfon C., Pickering B., Mubareka S. (2021). Evaluation of mobile real-time polymerase chain reaction tests for the detection of severe acute respiratory syndrome coronavirus 2. Sci. Rep..

[26] Ettinger J., Hofmann J., Enders M., Tewald F., Oehme R.M., Rosenfeld U.M., Ali H.S., Schlegel M., Essbauer S., Osterberg A. (2012). Multiple synchronous outbreaks of Puumala virus, Germany, 2010. Emerg Infect. Dis..

[27] Song J.W., Moon S.S., Gu S.H., Song K.J., Baek L.J., Kim H.C., Kijek T., O’Guinn M.L., Lee J.S., Turell M.J. (2009). Hemorrhagic fever with renal syndrome in 4 US soldiers, South Korea, 2005. Emerg Infect. Dis..

[28] Kim H.-C., Kim W.-K., No J.S., Lee S.-H., Gu S.H., Chong S.-T., Klein T.A., Song J.-W. (2018). Urban Rodent Surveillance, Climatic Association, and Genomic Characterization of Seoul Virus Collected at US Army Garrison, Seoul, Republic of Korea, 2006–2010. Am. J. Trop. Med. Hyg..

[29] Kim W.K., No J.S., Lee S.H., Song D.H., Lee D., Kim J.A., Gu S.H., Park S., Jeong S.T., Kim H.C. (2018). Multiplex PCR-Based Next-Generation Sequencing and Global Diversity of Seoul Virus in Humans and Rats. Emerg Infect. Dis..

[30] Lee G.Y., Kim W.K., Park K., Lee S.H., Hwang J., No J.S., Cho S., Lee D., Song D.H., Gu S.H. (2020). Phylogeographic diversity and hybrid zone of *Hantaan orthohantavirus* collected in Gangwon Province, Republic of Korea. PLoS Negl. Trop. Dis..

[31] Irwin D.M., Kocher T.D., Wilson A.C. (1991). Evolution of the cytochrome *b* gene of mammals. J. Mol. Evol..

[32] Kim H.-C., Kim W.-K., Klein T.A., Chong S.-T., Nunn P.V., Kim J.-A., Lee S.-H., No J.S., Song J.-W. (2017). Hantavirus surveillance and genetic diversity targeting small mammals at Camp Humphreys, a US military installation and new expansion site, Republic of Korea. PLoS ONE.

[33] Ronquist F., Teslenko M., van der Mark P., Ayres D.L., Darling A., Hohna S., Larget B., Liu L., Suchard M.A., Huelsenbeck J.P. (2012). MrBayes 3.2: Efficient Bayesian phylogenetic inference and model choice across a large model space. Syst. Biol..

[34] Kumar S., Stecher G., Tamura K. (2016). MEGA7: Molecular Evolutionary Genetics Analysis Version 7.0 for Bigger Datasets. Mol. Biol. Evol..

[35] Yasuda S.P., Yoshimatsu K., Koma T., Shimizu K., Endo R., Isozumi R., Arikawa J. (2012). Application of truncated nucleocapsid protein (N) for serotyping ELISA of murinae-associated hantavirus infection in rats. J. Vet. Med. Sci..

[36] Lee H.W., Baek L.J., Johnson K.M. (1982). Isolation of Hantaan virus, the etiologic agent of Korean hemorrhagic fever, from wild urban rats. J. Infect. Dis..

[37] Dupinay T., Pounder K.C., Ayral F., Laaberki M.H., Marston D.A., Lacote S., Rey C., Barbet F., Voller K., Nazaret N. (2014). Detection and genetic characterization of Seoul virus from commensal brown rats in France. Virol. J..

[38] Hu D., Hao L., Zhang J., Yao P., Zhang Q., Lv H., Gong X., Pan X., Cao M., Zhu J. (2015). Development of reverse transcription loop-mediated isothermal amplification assays to detect Hantaan virus and Seoul virus. J. Virol. Methods.

[39] Kang X., Li Y., Liu H., Lin F., Cai X., Sun T., Chang G., Zhu Q., Yang Y. (2010). A duplex real-time reverse transcriptase polymerase chain reaction assay for detecting western equine and eastern equine encephalitis viruses. Virol. J..

[40] Huhtamo E., Hasu E., Uzcategui N.Y., Erra E., Nikkari S., Kantele A., Vapalahti O., Piiparinen H. (2010). Early diagnosis of dengue in travelers: Comparison of a novel real-time RT-PCR, NS1 antigen detection and serology. J. Clin. Virol..

[41] Mackay I.M. (2004). Real-time PCR in the microbiology laboratory. Clin. Microbiol Infect..

[42] Naslund J., Kerner A., Drobni P., Bucht G., Evander M., Ahlm C. (2011). Detection of Puumala and Rift Valley Fever virus by quantitative RT-PCR and virus viability tests in samples of blood dried and stored on filter paper. J. Virol. Methods.

[43] Tomaszewicz Brown A., McAloose D., Calle P.P., Auer A., Posautz A., Slavinski S., Brennan R., Walzer C., Seimon T.A. (2020). Development and validation of a portable, point-of-care canine distemper virus qPCR test. PLoS ONE.

[44] Grubaugh N.D., Ladner J.T., Lemey P., Pybus O.G., Rambaut A., Holmes E.C., Andersen K.G. (2019). Tracking virus outbreaks in the twenty-first century. Nat. Microbiol..

[45] Houldcroft C.J., Beale M.A., Breuer J. (2017). Clinical and biological insights from viral genome sequencing. Nat. Rev. Microbiol..

[46] Carroll M.W., Matthews D.A., Hiscox J.A., Elmore M.J., Pollakis G., Rambaut A., Hewson R., Garcia-Dorival I., Bore J.A., Koundouno R. (2015). Temporal and spatial analysis of the 2014-2015 Ebola virus outbreak in West Africa. Nature.

[47] De Jesus J.G., Giovanetti M., Rodrigues Faria N., Alcantara L.C.J. (2019). Acute Vector-Borne Viral Infection: Zika and MinION Surveillance. Microbiol. Spectr..

[48] Perez-Sautu U., Gu S.H., Caviness K., Song D.H., Kim Y.J., Paola N.D., Lee D., Klein T.A., Chitty J.A., Nagle E. (2020). A Model for the Production of Regulatory Grade Viral Hemorrhagic Fever Exposure Stocks: From Field Surveillance to Advanced Characterization of SFTSV. Viruses.

[49] Page A.J., Mather A.E., Le-Viet T., Meader E.J., Alikhan N.F., Kay G.L., de Oliveira Martins L., Aydin A., Baker D.J., Trotter A.J. (2021). Large-scale sequencing of SARS-CoV-2 genomes from one region allows detailed epidemiology and enables local outbreak management. Microb. Genom..

[50] Kim W.-K., Kim J.-A., Song D.H., Lee D., Kim Y.C., Lee S.-Y., Lee S.-H., No J.S., Kim J.H., Kho J.H. (2016). Phylogeographic analysis of hemorrhagic fever with renal syndrome patients using multiplex PCR-based next generation sequencing. Sci. Rep..

[51] Kim W.K., No J.S., Lee D., Jung J., Park H., Yi Y., Kim J.A., Lee S.H., Kim Y., Park S. (2020). Active Targeted Surveillance to Identify Sites of Emergence of Hantavirus. J. Infect. Dis..

[52] Kim W.K., Cho S., Lee S.H., No J.S., Lee G.Y., Park K., Lee D., Jeong S.T., Song J.W. (2020). Genomic Epidemiology and Active Surveillance to Investigate Outbreaks of Hantaviruses. Front. Cell Infect. Microbiol..

[53] Solà-Riera C., Gupta S., Ljunggren H.-G., Klingström J. (2019). Orthohantaviruses belonging to three phylogroups all inhibit apoptosis in infected target cells. Sci. Rep..

[54] Vulin J., Murri S., Madrieres S., Galan M., Tatard C., Piry S., Vaccari G., D’Agostino C., Charbonnel N., Castel G. (2021). Isolation and Genetic Characterization of *Puumala Orthohantavirus* Strains from France. Pathogens.

[55] Song G., Hang C., Liao H., Qiu X., Gao G., Du Y., Zhao J., Xu J., Kong B. (1982). Isolation of EHF-related agent from *Rattus norvegicus* captured from patients’ home in endemic areas of the mild type of hemorrhagic fever. Acta Microbiol. Sin..

[56] KITAMURA T., KOMATSU T., SUGIYAMA K., MORITA C., IMAIZUMI K., SHIGA S., AKAO Y., OYA A., TAKEDA H., ARIKAWA J. (1983). Isolation of virus causing hemorrhagic fever with renal syndrome (HFRS) through a cell culture system. Jpn. J. Med. Sci. Biol..

[57] Lee H.W. (1986). Global distribution and molecular biological characteristics of Hantaviruses. J. Bacteriol. Virol..

[58] Pilaski J., Ellerich C., Kreutzer T., Lang A., Benik W., Pohl-Koppe A., Bode L., Vanek E., Autenrieth I.B., Bigos K. (1991). Haemorrhagic fever with renal syndrome in Germany. Lancet.

[59] LeDuc J.W., Smith G.A., Johnson K.M. (1984). Hantaan-like viruses from domestic rats captured in the United States. Am. J. Trop. Med. Hyg..

