# Supplementary material for: A Portable Diagnostic Assay, Genetic Diversity, and Isolation of Seoul Virus from Rattus norvegicus Collected in Gangwon Province, Republic of Korea
Source: Pathogens. 2022 Sep 14;11(9):1047. doi: 10.3390/pathogens11091047 (PMC9503505; doi:10.3390/pathogens11091047)
Supplement: Supplementary file 1 [file pathogens-11-01047-s001.zip › Figure S1.pdf]

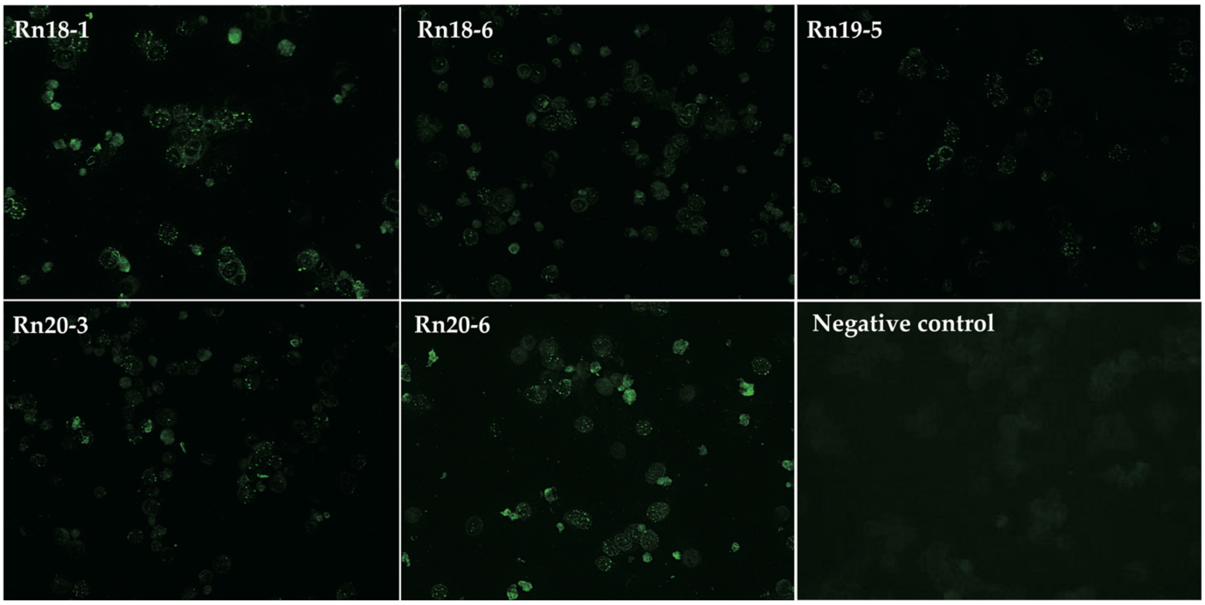

Figure S1. Detection of immunoglobulin G (IgG) antibodies against Seoul virus (SEOV) from heart fluids of *Rattus norvegicus* by indirect immunofluorescence assay (IFA). SEOV-infected Vero E6 cells were fixed on each slide. The heart fluids (1:2 dilution) of dead rats were used to detect anti-SEOV IgG by IFA, Rn18-1, Rn18-6, Rn19-5, Rn20-3, and Rn20-6. Negative control used was phosphate buffered saline (PBS). Rn, *R. norvegicus*.
