# Supplementary material for: A Portable Diagnostic Assay, Genetic Diversity, and Isolation of Seoul Virus from Rattus norvegicus Collected in Gangwon Province, Republic of Korea
Source: Pathogens. 2022 Sep 14;11(9):1047. doi: 10.3390/pathogens11091047 (PMC9503505; doi:10.3390/pathogens11091047)
Supplement: Supplementary file 1 [file pathogens-11-01047-s001.zip › Figure S2.pdf]

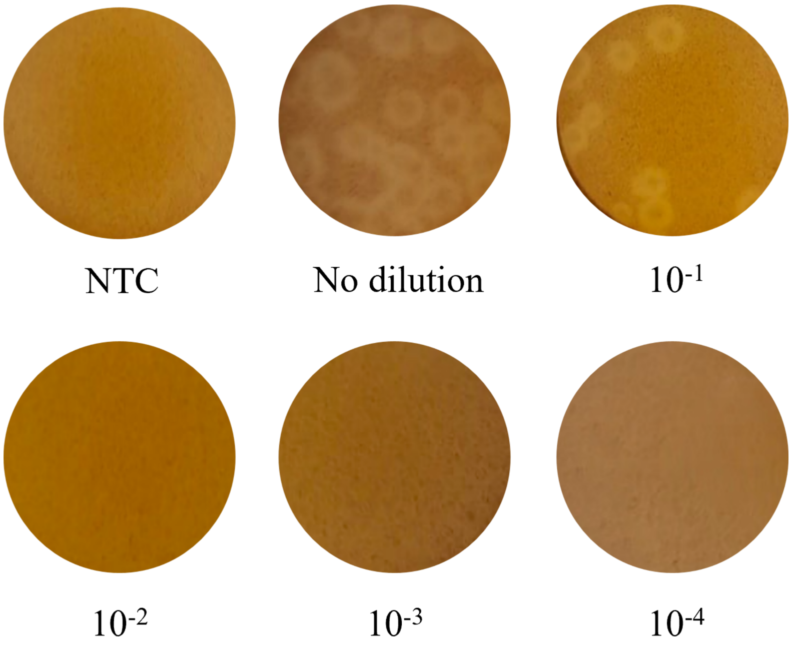

Figure S2. The plaque assay of SEOV Rn19-5 strain. SEOV Rn19-5 strain was quantified on 6-well plates with Vero E6 monolayers. Each well indicates different dilutions from top-left to right: NTC, no dilution, and dilutions at $1:10^1$, and bottom-left to right: dilutions at $1:10^2$, $1:10^3$, and $1:10^4$, respectively. The plaque was first observed at 9 days post infection. SEOV, Seoul virus; NTC, negative control.
