# Supplementary material for: A Portable Diagnostic Assay, Genetic Diversity, and Isolation of Seoul Virus from Rattus norvegicus Collected in Gangwon Province, Republic of Korea
Source: Pathogens. 2022 Sep 14;11(9):1047. doi: 10.3390/pathogens11091047 (PMC9503505; doi:10.3390/pathogens11091047)
Supplement: Supplementary file 1 [file pathogens-11-01047-s001.zip › Table S1.pdf]

Table S1. Trapping sites and GPS coordinates in this study.

Year	Trapping site	GPS coordinates
2016	Seoul Metropolitan City (Yongdu-dong)	37°34′42″ N 127°01′45″ E
	Seoul Metropolitan City (Anam-dong)	37°34′58″ N 127°01′45″ E
2017	Seoul Metropolitan City (Sinseol-dong)	37°34′32″ N 127°01′25″ E
	Seoul Metropolitan City (Yongdu-dong)	37°34′42″ N 127°01′45″ E
	Seoul Metropolitan City (Anam-dong)	37°34′58″ N 127°01′45″ E
2018	Chuncheon-si (Cheonjeon-ri)	37°56′34″ N 127°46′34″ E
2019	Gangneung-si (Haksan-ri)	37°42′51″ N 128°53′52″ E
2020	Seoul Metropolitan City (Anam-dong)	37°34′58″ N 127°01′45″ E
	Cheorwon-gun (Yuli-ri)	38°14′16″ N 127°12′3″ E
