# Supplementary material for: A Portable Diagnostic Assay, Genetic Diversity, and Isolation of Seoul Virus from Rattus norvegicus Collected in Gangwon Province, Republic of Korea
Source: Pathogens. 2022 Sep 14;11(9):1047. doi: 10.3390/pathogens11091047 (PMC9503505; doi:10.3390/pathogens11091047)
Supplement: Supplementary file 1 [file pathogens-11-01047-s001.zip › Table S2.pdf]

Table S2. Summary of mapped reads and depth of coverage for Seoul virus using next-generation sequencing in this study.

Sample	Total reads	Mapped reads/ Total reads (%)	L segment		M segment		S segment	
			Reads mapped to reference (%)	Depth of coverage	Reads mapped to reference (%)	Depth of coverage	Reads mapped to reference (%)	Depth of coverage
Rn18-1	2,057,486	1,023,268 (49.7)	117,462 (11.5)	2,076.0	426,755 (41.7)	12,578.9	479,051 (46.8)	27,824.2
Rn19-5	6,589,296	6,326,623 (96.0)	2,080,167 (32.9)	38,615.5	2,227,049 (35.2)	73,719.6	2,019,407 (31.9)	137,705.4
**Average (%)**	**4,323,391**	**3,674,946 (85.0)**	**1,098,815 (29.9)**	**20,345.7**	**1,326,902 (36.1)**	**43,149.2**	**1,249,229 (34.0)**	**82,764.8**

Rn, *Rattus norvegicus*; L, large; M, medium; S, small.

The depth of coverage was calculated using the number of mapped reads (read length × number of reads matching the reference/reference genome size).
